# New Generation of 3D Virtual Models with Perfusional Zones: Perioperative Assistance for the Best Pedicle Management during Robotic Partial Nephrectomy

**DOI:** 10.3390/curroncol30040304

**Published:** 2023-04-01

**Authors:** Daniele Amparore, Federico Piramide, Paolo Verri, Enrico Checcucci, Sabrina De Cillis, Alberto Piana, Gabriele Volpi, Mariano Burgio, Giovanni Busacca, Marco Colombo, Cristian Fiori, Francesco Porpiglia

**Affiliations:** 1 Department of Urology, San Luigi Gonzaga Hospital, University of Turin, 10043 Orbassano, Italy; 2 Department of Surgery, Candiolo Cancer Institute, FPO-IRCCS, 10060 Candiolo, Italy

**Keywords:** three-dimensional imaging, robotic surgery, nephron-sparing surgery, kidney cancer, surgical anatomy

## Abstract

Selective clamping during robot-assisted partial nephrectomy (RAPN) may reduce ischemia-related functional impairment. The intraoperative use of 3D-virtual models (3DVMs) can improve surgical planning, resulting in a greater success rate for selective clamping. Our goal is to introduce a new generation of 3DVMs, which consider the perfusion volumes of the kidney. Patients listed for RAPN from 2021 to 2022 were recruited. A selective clamping strategy was designed and intraoperatively performed based on the specifically generated 3DVMs. The effectiveness of selective clamping was evaluated using near-infrared-fluorescence imaging (NIRF) and 3DVM. Perfusion areas extensions were compared, and relevant preoperative characteristics were analyzed. In 61 of 80 (76.25%) cases, selective clamping was performed. The concordance between the 3DVM areas and the NIRF-enhanced areas was verified (k = 0.91). According to the distribution of perfused areas crossing the tumor, there were one, two, three, four, and five crossing areas, with relative perfusion rates of 13.75%, 35%, 32.5%, 13.75%, and 5%, respectively. Lesion diameter and mesorenal location were the only factors related to a higher number (>3) of perfusion volumes crossing the lesion. The implementation of mathematical algorithms to 3DVMs allows for precise estimation of the perfusion zone of each arterial branch feeding the organ, leading to the performance of safe and effective pedicle management planning.

## 1. Introduction

Owing to the introduction of several new technologies and techniques in actual surgical practice, it is currently possible to perform more tailored and less invasive surgeries. Therefore, this evolution of surgical environment has been called “precision surgery” [1], and its aim is the continuous improvement of both oncological and functional outcomes.

To obtain a precise, tailored surgical procedure, the standard bidimensional imaging might sometimes be suboptimal since it requires a building-in-mind process by the surgeon to recreate the real aspects of the target organ in a three dimensional environment [2]. Therefore, three-dimensional virtual models (3DVMs) have been developed and introduced, becoming very attractive in the urological field and especially in renal surgery [3]. By increasing surgeons’ awareness of tumor complexity and renal vascularization [4,5,6], 3DVMs have been shown to increase the success rate of selective clamping and to minimize the renal functional impairment while improving the management of higher-complexity tumors [7,8,9]. 

Although it has shown promising results, 3DVM-based surgical planning sometimes the fails intraoperatively. Indeed, even if the planning is guided by 3DVMs, allowing the surgeon to simulate different selective clamping options and to perceive the relative areas of devascularization and consequent ischemia, the planned clamping strategy (also if checked via near-infrared fluorescence guidance—NIRF) can be insufficient during surgery to guarantee an appropriate avascular resection plane, forcing the surgeon to switch the strategy to main renal artery clamping. The modification of the intraoperative vascular management approach can be explained considering that, when we plan selective clamping, we consider only the direction of the arterial branches toward the kidney surface instead of the perfused parenchymal volumes fed by these vessels and their boundaries.

To overcome this limit and precisely estimate the perfusion renal zones, we further implemented our models by introducing a mathematical algorithm able to identify the different perfusion volumes of the organ. The aim of the study was to test the accuracy of these new 3DVMs in guiding the clamping strategy and assessing whether there are factors influencing the tumor location relative to these perfusion volumes.

## 2. Materials and Methods

We prospectively enrolled all patients with a radiological diagnosis of an organ-confined solitary renal tumor scheduled for robot-assisted partial nephrectomy (RAPN) with intraoperative imaging guidance at our center from January 2021 to May 2022.

Informed consent was obtained, and the trial was performed in accordance with best clinical practice recommendations. According to Italian law, no formal institutional review board or ethical committee permission was required (Agenzia Italiana del Farmaco Guidelines for Observational Studies, 20 March 2008). Evidence of anatomical anomalies, such as a horseshoe-shaped or ectopic kidney, was used as an exclusion criterion. Patients who had preoperative imaging that was insufficient for producing a 3DVM (such as CT with a >3-mm acquisition interval of the slices or unsatisfactory difference of enhancement among the phases) or who had imaging older than three months were also excluded.

According to the previous description, Medics Srl © (www.medics3d.com, Turin, Italy) developed the 3DVMs for the cases starting from CT scans [7]. To estimate the perfusion volumes of the organ, the 3DVMs were then implemented by Medics bioengineers with an internal proprietary algorithm, based on the Voronoi diagram, a Euclidean distance-based mathematical method used to determine vascular dominating volumes in other organs, as previously described by Wang et al. [10]. 

By dividing the healthy parenchyma into anatomical sections based on the morphology and proximity of the arteries, the 3DVM perfusion zones were evaluated. A centerline was retrieved from the artery model using the commonly used vascular modeling toolkit (VMTK) libraries [11]. The 1D vascular tree spatial representation used to represent the vascular branch course inside the organ was represented by centerline points. According to a proximity requirement, each centerline point (also known as a seed) can be connected to organ parenchyma voxels. The nearest squared Euclidian distance from a seed point on the artery centerline was computed for each kidney voxel.

**Figure 1:**
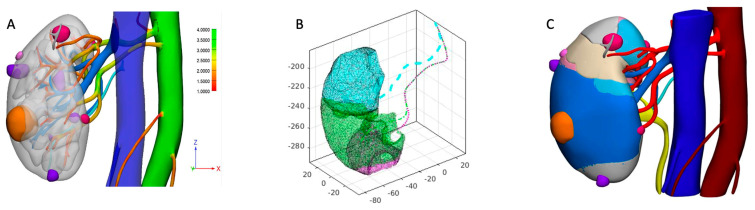
Description of perfusion 3DVM building process. (**A**) A standard 3DVM is built, and vessels of interest are identified. (**B**) For each portion of parenchyma, the closest distance from each artery is calculated, and perfusion regions are automatically built. (**C**) Finally, the 3D virtual perfusion model is refined and finalized as a 3D navigable PDF, allowing the surgeon to appreciate the different perfusion regions of the organ, the tumor (orange), and cyst (purple and violet) locations.

Precise artery segmentation is essential to accurately estimate kidney perfusion regions: the higher the detail of smaller branching, the greater the accuracy of perfusion zone subdivisions. This method allows for determining the final perfusion volumes related to the clampable blood vessels during surgery (Figure 1). All procedures were performed with transperitoneal access by a single experienced surgeon. 

Before each case, the surgeon studied the 3DVMs and planned the surgical strategy accordingly. Moreover, he precisely defined its clamping strategy (i.e., selective or global) based upon the information given by the 3DVMs. Intraoperatively, the model was available on demand electronically (using an tablet or a PC) or in an augmented reality fashion [12,13,14,15]. 

After kidney exposure, under 3DVM guidance, the main renal artery was identified and dissected, isolating the segmental branches entering the renal sinus or some accessory and aberrant arteries eventually reported by the 3DVM. When possible, depending on the specific case anatomy, pedicle dissection was conducted in a centripetal fashion, from the sinusal rim toward the aorta, starting with isolation of the portion of the segmental arteries facing the sinusal orifice and often limiting the isolation to this order of vessels, following the information given by the 3DVM. Once the renal pedicle was dissected, the renal mass was exposed or marked on the kidney’s surface (if totally endophytic) under augmented reality (AR) guidance and ultrasound (US) confirmation [8]. Then, the arteries preoperatively identified under 3DVM assistance and the branches feeding the perfusion zones close to the renal mass were clamped with Bulldogs, performing selective clamping. When global ischemia was planned after 3DVM consultation, the main artery was clamped, while no clamping was performed when a clampless procedure was planned.

**Figure 2:**
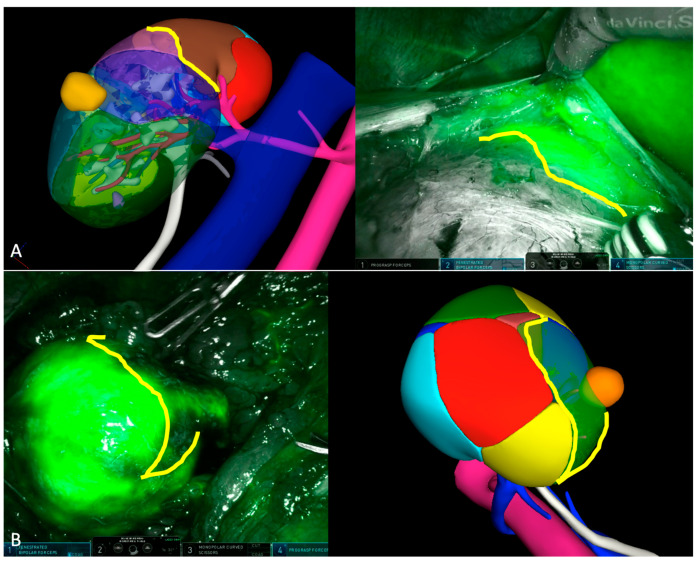
Intraoperative check of selective clamping strategy using near-infrared fluorescence imaging. (**A**) The boundary lines of perfused parenchyma perfectly reproduce the margins of perfusion zones of the 3DVM at the level of the upper pole of the right kidney. (**B**) In this second case, the boundary lines depicted via NIRF show similar orientation with those of the 3DVM.

NIRF became the indicator of proper vascular control in those instances treated with selective clamping. The intravenous injection of a indocyanine green (ICG) solution (0.1–0.5 mg/mL/kg) allowed for the precise identification of the boundaries and the shapes of the different perfusion regions of the kidney, permitting visualization of portions still receiving blood flow as bright green areas, while clamped vessels were gray [16,17] (Figure 2A,B).

We prospectively gathered demographic information for each patient, including age, body mass index (BMI), American Society of Anesthesiologists (ASA) score, and comorbidities as reported in the Charlson’s comorbidity index [18]; clinical tumor size, side, location, and complexity according to the PADUA score [19]; and perioperative information (including management of the renal pedicle, type, and duration of ischemia), pathological information (including stage according to TNM), and postoperative complications as classified by the Clavien–Dindo system [20].

**Figure 3:**
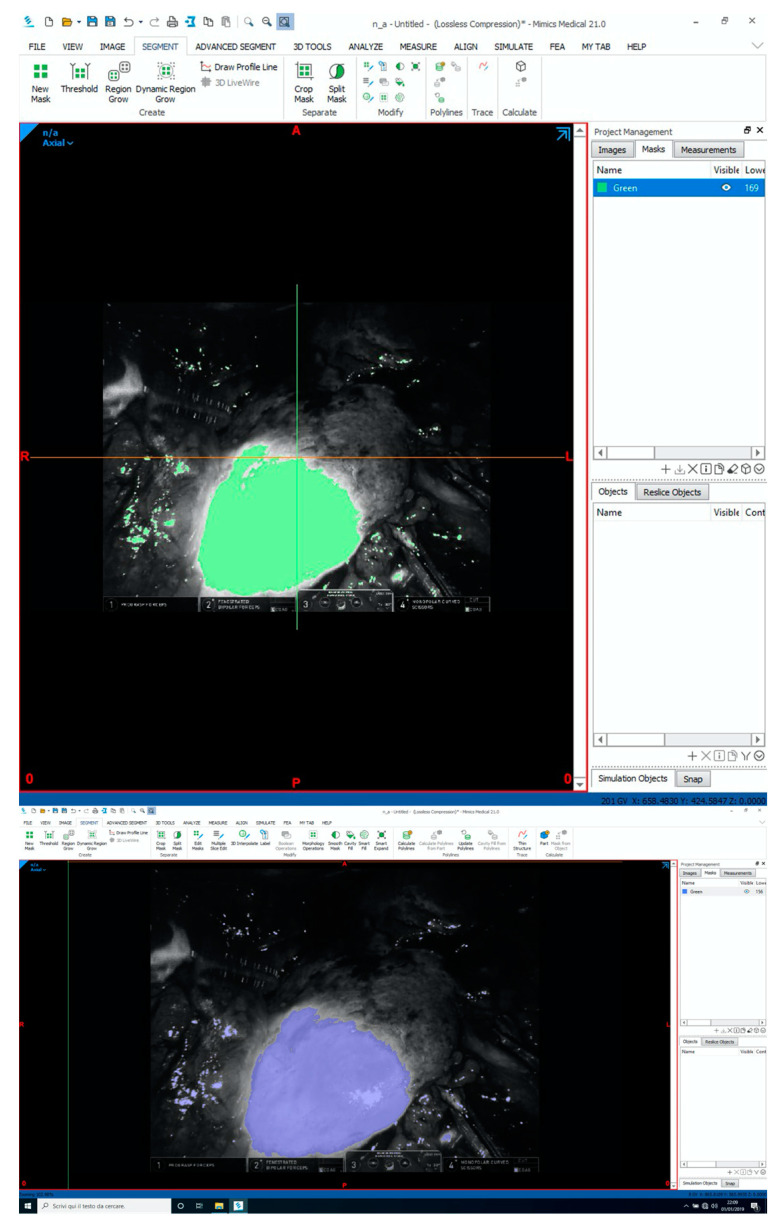
Using Mimics 21.0 (Materialise, Leuven, Belgium), it is possible to calculate the concordance between 3DVM regions and reality, measuring the extension of the 3DVM areas overlaid on the intraoperative stream and comparing them with the ICG-enhanced area, perceived as a group of pixels of the same color, from the recorded surgical image.

Per the study purpose, to demonstrate the concordance between virtual and real perfusion volumes, superimposition of the 3DVMs over the real anatomy was performed on bidimensional images recorded at the time of surgery. To superimpose the intraoperative ICG view with the 3D model, we used Mimics software, version 21.0 (Materialise, Leuven, Belgium), uploading a screen extrapolated from the intraoperative video. The areas maintaining perfusion on the virtual model were selectively overlaid on the endoscopic image during the ICG injection to verify the concordance between the boundary lines (Figure 2A,B). Moreover, their relative areas were automatically calculated using dedicated software, which could perceive pixels of the same color from a bidimensional image (Figure 3).

After evaluating each 3DVM, the total number of perfusion regions was recorded, as well as their relative volumes. Last, the position of each renal mass was assessed in proportion to the number of perfused regions it crossed.

Using the Mann–Whitney test for continuous variables and Fisher’s exact test for categorical ones, patient characteristics were examined. Frequencies and proportions were given as percentages, and all results for continuous variables were expressed as medians (interquartile range).

The extent of the vascularized areas calculated from the endoscopic NIRF video bidimensional frames and their virtual overlaid counterparts were compared via the k-Cohen test. Finally, a multivariate regression model (MLR) was fitted to assess tumor factors potentially related to the number of perfusion volumes crossed by the lesion. In particular we tested whether tumor variables, such as lesion size, PADUA score, tumor histology, stage, grade, sarcomatoid differentiation, necrosis, and microvascular invasion, were correlated with a number of tumor perfusion regions greater than three. All data were analyzed using Jamovi software, version 2.3.

## 3. Results

During the enrollment phase, 3DVM-assisted RAPN was performed on 80 patients. Table 1 reports patient demographics and preoperative features. The median (interquartile range) age was 64 years old, while the median (IQR, interquartile range) BMI and Charlson’s comorbidity index were 24.3 (22.8–27.9) and 2 (2–3), respectively. The median tumor size was 35.5 mm, with 57.5% classified as cT1a masses. The majority of lesions were right sided (61.25%) and located at the posterior face of the organ (52.5%). The median PADUA score was 8 (7–9). Among the 80 renal masses considered, 41.25% (33/80) were classified as “low complexity”, 40% (32/80) as “intermediate”, and 18.75% (15/80) as “highly complex”.

Table 2 shows perioperative and pathological information.

While 49 patients had cognitive intraoperative assistance, 31 patients underwent 3DVM-assisted RAPN with AR guidance.

Selective clamping was used to manage the vascular pedicle in 61 (76.25%) patients, main artery clamping was performed in 12 (15%) patients, and clampless surgery was chosen in seven (8.75%) patients.

For both global and selective clamping, the median (IQR) ischemia times were 17 (15–25) and 16 (12–20) minutes, respectively. In 28 cases (35%), pure enucleation was performed. In 18.75% of the procedures, we recorded a violation of the collecting system.

Without varying depending on the hilar clamping technique, the median (IQR) operating time and estimated blood loss (EBL) were 95 min (80–130) and 265 mL (150–300), respectively.

No conversions to radical nephrectomy occurred, and there was only one (1.25%) intraoperative complication (i.e., focal violation of an arterial aneurysm at the level of the retro-pyelic artery, handled intraoperatively with a dedicated suture). The overall postoperative complication rate was 11.25% (9/80), with a Clavien >2 complication rate of 2.5%. Two (2.5%) positive surgical margins (PSMs) were recorded.

Based on 3DVMs, Table 3 reports the cohort’s perfusional kidney and tumor features.

Eight perfusion volumes on average (IQR) per kidney (7–10) were recorded. According to 3DVMs, the distribution of perfused regions crossing the tumor was as follows: 13.75%, 35%, 32.5%, 13.75%, and 5% for one, two, three, four, and five crossing areas, respectively.

Moreover, the median (IQR) global volume of kidneys, given by the sum of all its perfusion zones was 113.8 cm^3^ (94.9–137.1), with a median volume of perfusion volumes contacting the tumor of 48.2 cm^3^ (33.4–74.1) and a median volume of regions not in contact with the renal mass of 65.6 cm^3^ (39.8–81.6).

Considering the intraoperative assessment with ICG, the concordance between the bidimensional NIRF-enhanced areas and their corresponding 3DVM areas, automatically calculated on endoscopic video frames in cases that underwent selective clamping, was confirmed by the Cohen-k test, with a k = 0.91.

At MLR, larger tumor sizes (OR 1.036, CI 95% 1.0003–1.073; *p* 0.048) and a mesorenal location (OR 3.291, CI 95% 1.1288–9.593; *p* = 0.029) were the only two factors related to a higher number (>3) of perfusion volumes crossing the lesion, while the other biological variables (histology, ISUP grade, sarcomatoid differentiation, necrosis, and microvascular invasion) did not reach statistical significance.

## 4. Discussion

We have already investigated how surgeons can benefit from 3DVMs during RAPN, showing how this technique can prevent the healthy renal remnant from global ischemia injury [7,8]. Furthermore, the advantages of 3DVM-guided minimally invasive PN have been also clearly demonstrated by a recent systematic review of comparative studies [21]. The use of 3DVMs allowed for better surgical outcomes in reducing the global ischemia rate, opening of the urinary system, and blood loss and in increasing the adoption of enucleation techniques. Additionally, the latest evidence shows that 3DVMs may have a preventing role against the decline of renal function (measured by renal scan) after minimally invasive PN [9].

Although a virtual replica of the genuine organ has helped, up to now, the management of the vascular pedicle has mostly been empirical, requiring intraoperative change of the clamping technique in situations in which unanticipated hemorrhage from the resection occurred [22]. This proof is consistent with some anatomical investigations showing that the distribution of arterial branches in the renal artery vasculature is highly variable (for example, with usually more than one artery supplying the same pole), making it impossible to schematize it using the traditional Graves classification [23,24,25] for more than half of our patients. 

After taking everything into account, for the present study, we created a new generation of 3DVMs using a mathematical approach, in which the renal parenchyma is made up of the perfusion zones of each segmental artery entering the tissue, overcoming the empirical definition of these vessels proposed by Graves. As a result, we evaluated its effectiveness in enabling precise selective clamping and devascularization of the perilesional parenchymal volume. We also examined its accuracy in defining each perfusion zone, comparing its relative real intraoperative counterpart.

One of the first findings to be emphasized is the huge variability in the number of regions identified by the algorithm across the whole cohort. Indeed, they ranged from four to 13, significantly higher than the five regions described by Graves. Similarly, the number of regions contacting the tumor was also different depending on each specific case.

Although 48.5% of the virtual reconstructions revealed one or two perfusion regions in touch with the lesion’s surface, 33% showed tumors fed by three regions and 18.5% by four or more. This evidence can be partly explained considering that the information given to the software by the CT scan images can be influenced by the contrast enhancement distribution, quality of the images, or time of acquisition, emphasizing how the renal anatomy can be different across the population of patients harboring a renal mass.

Another aspect to be considered when examining our perfusion 3DVMs is the assessment of each kidney perfusion volume. As shown by our results, the median volume of the perfusion regions in contact with the tumor was 42% of the median of the whole kidney (113.8 cm^3^/48.2 cm^3^), ranging between 30% and 50% of the total volume of the organ, considering the 25th and 75th quartiles. This information needs to be taken in account when the surgeon plans the renal pedicle management strategy. If we want to gain a bloodless resection bed while maintaining as much as possible the vascularization of the kidney, we need to expose at least one-third of it to ischemia. Moreover, such a parenchymal zone will suffer not only due to the temporary ischemic injury given by the absence of blood flow but also due to the loss of renal units close to the tumor’s pseudocapsule due to the resection of the renal mass itself and to the ischemic damage caused by suturing of the resection bed. All these factors considered, the balance between the risks and the benefits of a selective clamping strategy can be more consciously assessed, especially in cases of patients with a solitary kidney or chronic kidney disease [26,27]. Moreover, it could be useful to set the proper selective clamping strategy itself, depending on the patient-specific vascular anatomy. Based on 3DVMs with perfusion zones, it is possible to plan the clamping of two or three segmental arteries feeding the tumor region or only the prepyelic or the retropyelic branch of the main renal artery, balancing the trade-off between the amount of healthy tissue spared from ischemia and the feasibility of selective clamping at the level of the renal sinus or involving multiple segmental branches.

As a result, in our case series, there was a high rate of effective selective clamping performed under the supervision of the perfusional-regions-3DVMs (76.25%). This high rate of effective selective clamping, together with the adoption of a clampless strategy in some cases (8.75%), allowed for limiting as much as possible the use of global clamping (15%). Moreover, the use of these new virtual models in the intraoperative assistance of RAPN was safe, as demonstrated by the low rate of intra- and post-operative complications, as well as the rate of positive surgical margins. All these outcomes are in line with the concept of an adequate clamping strategy of the renal pedicle, not leading to uncontrolled bleeding potentially responsible for complications during or after the surgery or hiding the cleavage plane of the tumor potentially responsible for a positive surgical margin at the level of the resection bed [28,29,30].

The accuracy of the perfusion volumes-3DVMs was also demonstrated by their comparison with intraoperative evidence shown by ICG injection. The bidimensional images superimposed on the areas maintaining perfusion on the 3DVMs over the endoscopic image, recorded at the surgery time via ICG injection, demonstrated a high rate of concordance in terms of boundary lines and areas, with a Cohen-k coefficient of 0.91.

Last, a multivariate regression model was created to evaluate the factors potentially related to the number of perfusion volumes contacting the tumor. This analysis was performed to consider the characteristics of the environment in which the lesion grows. Together with the anatomical features of the lesion and its location in the kidney (i.e., size, polar location, growth pattern, and renal face), the histology and the grade of the tumor were also assessed. The multivariate analysis found a significant correlation between the higher number of tumors contacting perfusion volumes and the tumor size and mesorenal location. This evidence is explainable, considering that the larger the tumor is in size, the higher the probability is of having a wide resection bed intercepting different perfusion volumes. Similarly, mesorenal tumors more often grow in contact with a higher number of perfusion volumes. Interestingly, the kidney face on which the tumor is located was not a factor related to the number of perfusion volumes intercepted. This evidence confirms the previous finding, in contrast with the traditional anatomical principles of vascularization established by Graves [23,24,25]. Indeed, the growth of a tumor on the posterior or anterior face does not seem to relate to its possibility of being in contact with few or many volumes, with their boundaries being highly variable across the patients.

Notwithstanding this interesting evidence, our study is not free of limitations. First, the real impact of our technology could be difficult to estimate considering the lack of a control group. Furthermore, all our surgeries were performed by a very experienced robotic surgeon, and therefore, the advantages of our technology might be overestimated, especially in terms of ischemia time, complications, and positive surgical margin rate, and could also limit the generalizability of our findings. Last, since this technology is new and rapidly evolving, its costs are difficult to define and might limit its diffusion in daily practice.

To the best of our knowledge, this study is the first to demonstrate the accuracy and efficacy of this new generation of 3DVMs. With the implementation of a mathematical algorithm in the production of a virtual model, it is possible to have a virtual representation of the kidney composed of all its perfusion zones, pushing forward the limit of the information given by the human eye and making a significant contribution to the current anatomical principles of kidney vasculature. 

Its use in the intraoperative setting allows the surgeon to follow step by step the strategy set before the intervention with the consultation of the perfusion regions-3DVM, performing precise and unfailing selective clamping and giving the proper frame of mind to deal with the tumor resection and suture phases without distress due to the time spent under global ischemia of the kidney. In fact, considering the ischemia matter, we can argue that time and extension are inversely proportional for the postoperative global renal function: if the ischemic injury affects the whole kidney, it is necessary to reduce as much as possible the time of exposure, while if the portion of the organ injured is limited, the minimization of exposure time becomes less significant.

## 5. Conclusions

This study emphasizes how technology, supported by the implementation of mathematical software, does not fail to enhance the lines traced by the anatomy. With these new virtual reconstructions, we are daily one step closer to connecting surgical anatomy to microscopy. All these innovations will lead to a super-tailored procedure, allowing us to spare the maximum functional tissue, minimizing the extension and duration of ischemia with the highest level of safety possible.

## Figures and Tables

**Table 1:** Patients’ characteristics.

Patients’ Characteristics	Perfusion Volumes 3DVM-RAPN n = 80
Age, yrs median (IQR)	64 (57–71)
BMI (kg/m^2^), median (IQR)	24.3 (22.8–27.9)
CCI, median (IQR)	2 (2–3)
ASA score, median (IQR) • <2 • >2	35 (73) 13 (27)
CT lesion size (mm) median (IQR)	35.5 (25.8–48.5)
Clinical stage, n (%) • cT1a • cT1b	46 (57.5) 34 (42.5)
Kidney face location, n (%) • Anterior face • Posterior face	38 (47.5) 42 (52.5)
Tumor side, n (%) • Right • Left	49 (61.25) 31 (38.75)
Tumor location, n (%) • Upper pole • Mesorenal • Lower pole	8 (10) 56 (70) 16 (20)
Tumor growth pattern, n (%) • >50% Exophytic • <50% Exophytic • Endophytic	41 (51.25) 24 (30.0) 15 (18.75)
Kidney rim location, n (%) • Lateral margin • Medial margin	46 (57.5) 34 (42.5)
PADUA Score, median (IQR)	8 (7-9)
PADUA risk category, n (%) • 1 (PADUA < 8) • 2 (PADUA 8–9) • 3 (PADUA > 10)	33 (41.25) 32 (40.0) 15 (18.75)

ASA = American Society of Anesthesiologists; BMI = body mass index; CCI = Charlson’s comorbidity index; CT = computed tomography; 3DVM = three-dimensional virtual model; RAPN = robot-assisted partial nephrectomy; IQR: interquartile range.

**Table 2:** Perioperative variables.

Perioperative Variables	Perfusion Volumes 3DVM-RAPN n = 80	
Augmented reality procedures, n (%)		31 (38.75)
Operative time (min), median (IQR)		95 min (80–130)
Hilar clamping, n (%) • Global clamping • Selective clamping • clampless		12 (15) 61 (76.25) 7 (8.75)
Ischemia time (min), median (IQR) • Global ischemia • Partial ischemia		17 (15–25) 16 (12–20)
EBL (cc), median (IQR)		265 mL (150–300)
Extirpative technique, n (%) • Pure enucleation • Enucleoresection		28 (35) 52 (65)
Opened collecting system, n (%) • Yes • No		15 (18.75) 65 (81.25)
Intraoperative complications, n (%)		1 (1.25)
Postoperative complications, n (%)		9 (11.25)
Postoperative complications according to Clavien–Dindo, n (%)	>2	2 (2.5)
Length of stay (days), median (IQR)		5 (4–6)
Pathological stage. n. % • Benign • pT1a • pT1b • pT2 • pT3a		19 (23.75) 36 (45.0) 17 (21.25) 3 (3.75) 5 (6.25)
Pathological size (mm). median (IQR)		34 (25–48)
Positive surgical margin rate. n. %		2 (2.5)
Histopathological findings. n. % • Clear-cell carcinoma • Papillary • Chromophobe • Angiomyolipoma • Oncocytoma		41 (51.25) 15 (18.75) 5 (6.25) 4 (5.0) 15 (18.75)
ISUP grade. n. % • 1 • 2 • 3 • Not applicable		17 (21.25) 32 (40.0) 7 (8.75) 24 (32)

EBL = estimated blood loss; ISUP = International Society of Urological Pathology; 3DVM = three-dimensional virtual model; RAPN = robot-assisted partial nephrectomy; IQR: interquartile range.

**Table 3:** Perfusional kidney and tumor characteristics.

Variables	Perfusion-Volumes-3DVM RAPN, n = 80
Kidney’s perfusion regions, median (IQR)	8 (7–10)
Tumor’s perfusion regions, median (IQR)	3 (2–3)
Tumor’s perfusion regions, n (%) • 1 • 2 • 3 • 4 • 5	11 (13.75) 28 (35) 26 (32.5) 11 (13.75) 4 (5)
Volume of kidney’s perfusion regions, cm^3^, median (IQR)	113.8 (94.9–137.1)
Volume of perfusion regions in contact with the tumor, cm^3^, median (IQR)	48.2 (33.4–74.1)
Volume of perfusion regions non in contact with the tumor, cm^3^, median (IQR)	65.6 (39.8–81.6)

3DVM = three-dimensional virtual model; RAPN = robot-assisted partial nephrectomy; IQR: interquartile range.

## Data Availability

The data presented in this study are available on request from the corresponding author.
